# Beyond anosmia: olfactory dysfunction as a common denominator in neurodegenerative and neurodevelopmental disorders

**DOI:** 10.3389/fnins.2024.1502779

**Published:** 2024-10-30

**Authors:** Yu-Nan Chen, Johanna Katharina Kostka

**Affiliations:** Institute of Developmental Neuroscience, Center of Molecular Neurobiology, Hamburg Center of Neuroscience, University Medical Center Hamburg-Eppendorf, Hamburg, Germany

**Keywords:** olfaction, olfactory dysfunction, neurodegenerative disorders, neurodevelopmental disorders, Alzheimer’s disease, Parkinson’s disease, schizophrenia, autism spectrum disorder

## Abstract

Olfactory dysfunction has emerged as a hallmark feature shared among several neurological conditions, including both neurodevelopmental and neurodegenerative disorders. While diseases of both categories have been extensively studied for decades, their association with olfaction has only recently gained attention. Olfactory deficits often manifest already during prodromal stages of these diseases, yet it remains unclear whether common pathophysiological changes along olfactory pathways cause such impairments. Here we probe into the intricate relationship between olfactory dysfunction and neurodegenerative and neurodevelopmental disorders, shedding light on their commonalities and underlying mechanisms. We begin by providing a brief overview of the olfactory circuit and its connections to higher-associated brain areas. Additionally, we discuss olfactory deficits in these disorders, focusing on potential common mechanisms that may contribute to olfactory dysfunction across both types of disorders. We further debate whether olfactory deficits contribute to the disease propagation or are simply an epiphenomenon. We conclude by emphasizing the significance of olfactory function as a potential pre-clinical diagnostic tool to identify individuals with neurological disorders that offers the opportunity for preventive intervention before other symptoms manifest.

## Introduction

In humans, the sense of smell is often overlooked due to the dominance of vision and hearing in our daily lives. However, olfactory perception plays an important role in modulating cognition and emotions in healthy individuals (Richardson and Zucco, 1989; Sohrabi et al., 2012; Stevenson, 2013; Yahiaoui-Doktor et al., 2019). Olfactory performance decreases with age and correlates with cognitive abilities in the elderly (Attems et al., 2015; Murman, 2015; Uchida et al., 2020). Yet, olfactory impairments are also common symptoms of various neurodevelopmental and neurodegenerative disorders. Key olfactory functions—such as odor identification, odor discrimination, odor detection threshold, and odor memory processing—are frequently affected. Deficits in those olfactory functions can be readily assessed in humans using tests like Sniffin’ Sticks or the University of Pennsylvania Smell Identification Test (Doty et al., 1984; Hummel et al., 1997). Similarly, tests such as the buried pellet test, olfactory habituation/dishabituation tests, and olfactory preference/avoidance assays can be employed to evaluate olfactory impairments in mouse models of neurodevelopmental and neurodegenerative diseases, providing a valuable link between animal studies and human conditions (Yang and Crawley, 2009; Meyer and Alberts, 2016). The prevalence of olfactory deficits as a symptom of numerous neurodevelopmental and neurodegenerative diseases is striking. For instance, approximately 90% of patients with Alzheimer’s Disease (AD) and Parkinson’s disease (PD) exhibit olfactory impairments (Doty et al., 1988; Doty, 2017). Crucially, deficits in odor detection and discrimination alongside pathological changes in olfactory brain areas, often precede cognitive and/or motor symptoms by years (Ross et al., 2008; Devanand et al., 2010). Similarly, a significant proportion of individuals with schizophrenia (SCZ) or autism spectrum disorder (ASD) experience problems with their sense of smell, often without being aware of it (Moberg et al., 1999; Corcoran et al., 2005; Bennetto et al., 2007; Koehler et al., 2018). Given the early onset of olfactory deficits in a broad spectrum of distinct neurological disorders, early damage to the olfactory system could play a significant role in the progression of these diseases. Thus, a deeper understanding of the mechanisms underlying olfactory dysfunction could provide valuable insights into disease progression. Additionally, screening for olfactory deficits may offer a means of pre-clinical diagnostics and intervention before more severe cognitive symptoms emerge.

## Tight anatomical and functional coupling between olfactory and cortical brain areas

**Figure 1:**
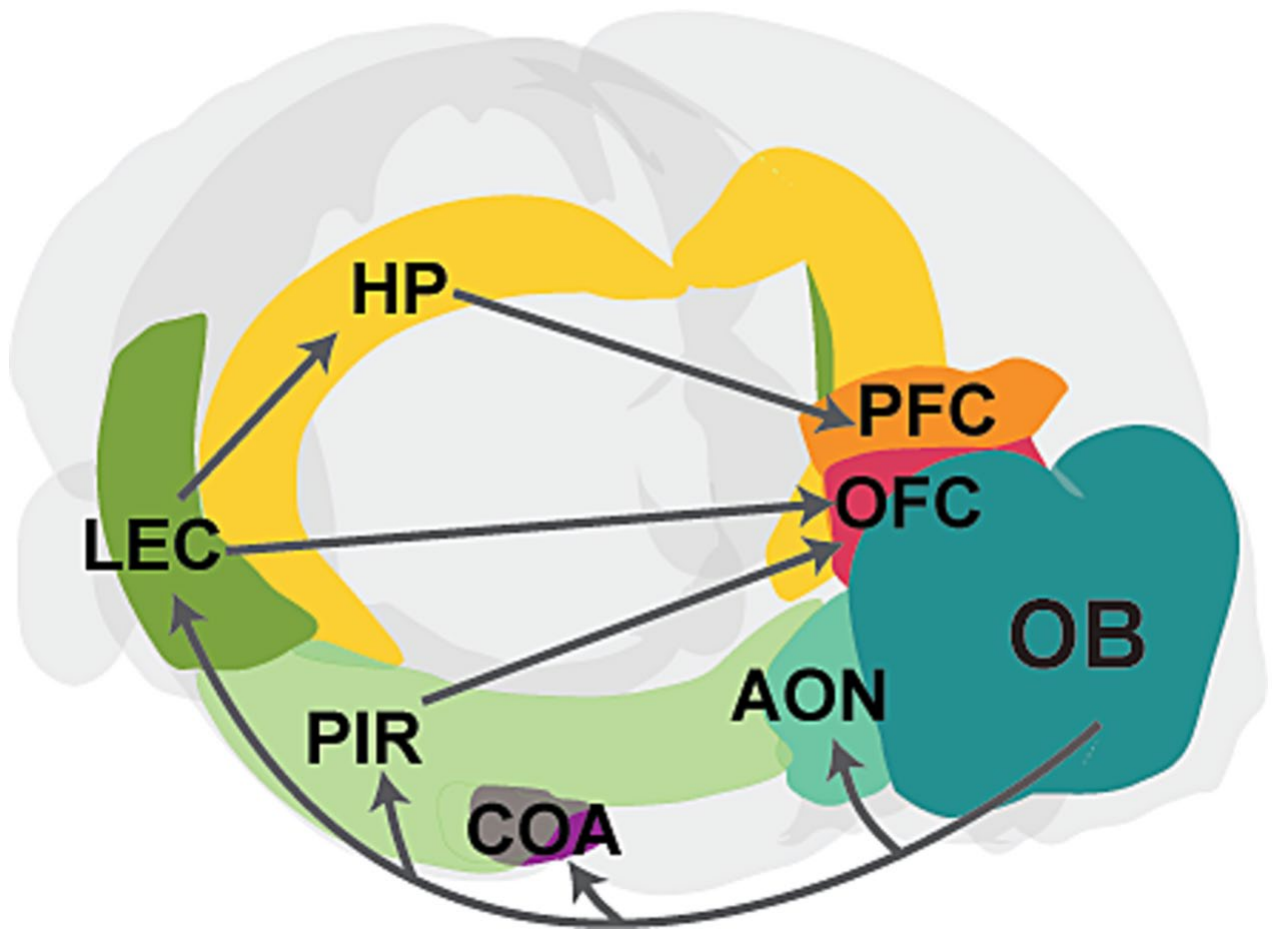
Schematic showing the main connectivity between the olfactory bulb and higher-order brain regions. The OB projects to primary cortical regions, including the anterior olfactory nucleus (AON), cortical amygdaloid nucleus (COA), piriform cortex (PIR), and lateral entorhinal cortex (LEC). Further, PIR and LEC send projections to higher-order cognitive regions, such as the hippocampus (HP), prefrontal cortex (PFC), and orbitofrontal cortex (OFC). The PFC also receives input from the HP. Gray arrows represent axonal projections, and individual areas are highlighted in different colors.

Olfactory sensing begins when odor molecules bind to diverse olfactory receptors on olfactory sensory neurons (OSNs) located within the olfactory epithelium (OE) in the nasal cavity (Zhang and Firestein, 2002). Sensory afferents from OSNs transmit excitatory signals to the olfactory bulb (OB), the main olfactory processing center. Mitral and tufted cells (M/TCs) in the OB relay this preprocessed information to various cortical and subcortical regions, such as the anterior olfactory nucleus (AON), piriform cortex (PIR), amygdala, and lateral entorhinal cortex (LEC; Sosulski et al., 2011; Igarashi et al., 2012; Imai, 2014). Olfactory cortical areas such as PIR and LEC subsequently project to higher-order brain areas, including the prefrontal cortex (PFC), orbitofrontal cortex (OFC), and hippocampus (HP), which are critical for cognitive functions (Witter et al., 2017; Figure 1). Unlike other sensory modalities, olfactory information bypasses the thalamus and directly connects to these higher-order brain regions. These direct connections are crucial for the processing of odor information. For instance, direct projections from LEC to HP are important for odor discrimination and memory (Leitner et al., 2016; Li et al., 2017), while connections from PIR to OFC are important for learning odor values (Wang et al., 2020). Moreover, slow respiration-driven oscillations in the OB modulate local field potentials in PIR, LEC, HP, and PFC (Zelano et al., 2016; Biskamp et al., 2017; Tort et al., 2018; Heck et al., 2022), and beta oscillations synchronize across olfactory and cognitive brain areas during working memory and decision-making, influencing task performance (Gourévitch et al., 2010; Mori et al., 2013; Igarashi et al., 2014; Rangel et al., 2016; Symanski et al., 2022). Studies have also shown that odor-induced fast oscillations in OB and PIR correlate with odor perception and discrimination (Beshel et al., 2007; Lepousez and Lledo, 2013; Yang et al., 2022).

OB networks are also strongly influenced by neuromodulatory inputs such as noradrenergic, serotonergic, and cholinergic inputs, which are involved in odor discrimination and odor learning (Linster and Fontanini, 2014; Brunert and Rothermel, 2021). Sparse dopaminergic (DA) input from the substantia nigra also terminates in the OB (Höglinger et al., 2015). A subpopulation of OB interneurons is both DA and GABAergic (Borisovska et al., 2013; Pignatelli and Belluzzi, 2017; Liu et al., 2019) and undergoes adult neurogenesis (Altman, 1969; Lazarini et al., 2014). These neurons modulate neurotransmitter release from OSNs and lateral inhibition within glomeruli (Hsia et al., 1999; Liu et al., 2013a; McGann, 2013) and are important for odor discrimination (Tillerson et al., 2006). Moreover, granule (GC) and periglomerular (PGC) interneurons in the OB, along with OSNs in the OE, are continuously generated throughout life (Murrell et al., 1996; Hahn et al., 2005; Batista-Brito et al., 2008; Lledo and Valley, 2016).

The olfactory system is anatomically and functionally interconnected with brain regions essential for cognitive processing. Importantly, pathological changes associated with neurodegenerative and neurodevelopmental disorders have been observed throughout the olfactory circuitry - from the OE and OB to primary olfactory cortices and downstream targets like LEC, HP, and PFC.

## Olfactory dysfunction in neurodegenerative disorders

Neurodegenerative disorders, like AD and PD, are characterized by progressive decline of cognitive and motor functions (Goedert and Spillantini, 2006; Wilson et al., 2023). Emerging evidence indicates that olfactory deficits—such as impaired odor detection and discrimination—manifest early in these diseases, preceding cognitive and motor symptoms by several years (Ross et al., 2008; Doty, 2017).

In AD, olfactory dysfunction correlates closely with the progression of cognitive decline (Roberts et al., 2016; Dintica et al., 2019; Papadatos and Phillips, 2023). Pathological features of AD include amyloid plaques (deposition of amyloid beta (Aβ) protein) and neurofibrillary tangles (aggregates of hyperphosphorylated tau proteins; Goedert and Spillantini, 2006; Ballard et al., 2011; Braak and Del Tredici, 2015). These pathological aggregations affect the OE and brain areas involved in odor processing, such as OB, AON, PIR, and LEC, often before clinical symptoms occur (Attems and Jellinger, 2006; Arnold et al., 2010; Murphy, 2019). Animal studies suggest that overexpression of the Aβ precursor protein causes olfactory deficits by progressive Aβ deposition, starting from the OE and expanding to the OB, PIR, entorhinal cortex (EC), and HP (Wesson et al., 2010; Wu et al., 2013). Similarly, in humans, areas like EC are among the first to be affected by AD pathology (Braak and Braak, 1991). Further, higher levels of phosphorylated tau (P-tau) in the OBs of AD patients correlate with MC loss, impaired dendro-dendritic inhibition, and diminished olfactory detection abilities before cognitive impairments emerged (Li et al., 2019a). Mouse models of Aβ pathology also show early olfactory deficits, alongside a loss of OSNs and decreased odor-evoked potentials in the OE, altered dendro-dendritic inhibition, and increased gamma oscillations in the OB, PIR, and LEC (Wesson et al., 2011; Xu et al., 2015; Li et al., 2019b; Chen et al., 2021b). These symptoms occur before Aβ plaque formation, suggesting that soluble Aβ might be responsible. In line with this, overexpression of a mutated human Aβ precursor protein in OSNs disrupts the glomerular axon targeting of those neurons and causes olfactory deficits before Aβ deposition forms in the OB (Cao et al., 2012). Similarly, injecting soluble Aβ oligomers into the OB damages the olfactory detection abilities of rodents (Alvarado-Martínez et al., 2013).

Similarly, olfactory dysfunction is an early and prominent non-motor symptom of PD (Ross et al., 2008; Doty, 2012, 2017; Haehner et al., 2019). PD patients score lower on the Sniffin’ Sticks Test compared to healthy controls (Haehner et al., 2007, 2009; Trentin et al., 2022), and brain areas such as the OB, AON, PIR and EC show early volume reductions (Wattendorf et al., 2009; Wang et al., 2011; Chen et al., 2014; Lee et al., 2014; Tanik et al., 2016). Characterized by *α*-synuclein aggregation forming Lewy bodies (Mezey et al., 1998), PD shows early pathological changes in the OB and AON (Braak et al., 2003). A transgenic mouse model of α-synuclein pathology confirms the prevalence of α-synuclein aggregation in the OB, AON, and PIR and shows reduced odor detection and diminished adult neurogenesis in the OB (Martin-Lopez et al., 2023). This aggregation is associated with increased odor-evoked gamma oscillations and altered neuronal firing in the OB (Chen et al., 2021a).

Thus, neurodegenerative disorders like AD and PD exhibit early olfactory deficits that coincide with the initial accumulation of pathological proteins in olfactory-related brain regions, before spreading to other parts of the brain.

## Olfactory dysfunction in neurodevelopmental disorders

Neurodevelopmental disorders, including SCZ and ASD, are characterized by atypical brain development and impaired cognitive, social, or motivation-related behaviors (Owen et al., 2016; Thye et al., 2018; Chini and Hanganu-Opatz, 2021). A prominent feature is impaired sensory processing (Schechter et al., 2003; Chang et al., 2014; Siper et al., 2021). In particular, reduced odor detection early in life, accompanied by anatomical and functional alterations in olfactory and higher-order cortical networks, is typical (Crow et al., 2020).

For instance, SCZ patients exhibit olfactory deficits and reduced OB, PIR EC, HP, and amygdala volumes, which precede the onset of cognitive deficits (Turetsky et al., 2000; Corcoran et al., 2005; Rupp et al., 2005; Nguyen et al., 2010, 2011; Kamath et al., 2018; Yang et al., 2021). Additionally, reduced olfactory-evoked potentials are associated with impaired odor identification in SCZ patients (Turetsky et al., 2003). Both genetic and environmental factors play a significant role in shaping the development of the olfactory system and are implicated in neurodevelopmental disorders. One prominent susceptibility factor for SCZ is the mutation of the Disrupted-in-Schizophrenia 1 (DISC1) gene, which is involved in various neuropsychiatric disorders (Blackwood et al., 2001; Chubb et al., 2008; Brandon et al., 2009) and is highly expressed in M/TCs (Schurov et al., 2004). DISC1 knockdown, combined with a prenatal environmental stressor - maternal immune activation (MIA) - leads to impaired oscillatory activity in the OB and reduced functional connectivity within olfactory-limbic networks of neonatal mice (Parbst et al., 2024; Xu et al., 2021).

In ASD, children often exhibit early olfactory deficits, including impaired odor identification along with reduced odor-evoked activity (Bennetto et al., 2007; Koehler et al., 2018). Like SCZ, both genetic and environmental factors contribute to the etiology of ASD. Genetic mutations, such as those affecting Shank proteins, involved in postsynaptic scaffolding, are prevalent in patients with ASD and are associated with olfactory deficits. Shank3 deficiency impairs odor detection, reduces odor-evoked potentials, and alters synaptic transmission in the OB and PIR (Drapeau et al., 2018; Ryndych et al., 2023; Mihalj et al., 2024). Moreover, mutation of the autism-related gene Tbr leads to smaller OBs, reduced numbers of OB interneurons, and abnormal dendritic morphology of MCs (Huang et al., 2019). Environmental factors like MIA, which largely increases the risks for both ASD and SCZ (Hartung et al., 2016; Schepanski et al., 2022; Dutra et al., 2023; Godavarthi et al., 2024), can impair adult neurogenesis in the OB and contribute to decreased olfactory discrimination abilities (Liu et al., 2013b).

Thus, olfactory dysfunctions accompanied by pathophysiological changes in brain areas associated with olfaction, are frequently observed in neurodevelopmental disorders.

## Shared structural and functional alterations and their underlying mechanisms in neurodegenerative and neurodevelopmental disorders

Olfactory deficits in neurodevelopmental and neurodegenerative disorders often coincide with structural alterations across brain regions involved in olfactory processing. For example, reduced OB volume has been documented in AD (Thomann et al., 2009b; Thomann et al., 2009a), PD (Wattendorf et al., 2009; Wang et al., 2011), and SCZ (Turetsky et al., 2000; Nguyen et al., 2011; Yang et al., 2021). This reduction in OB volume might be caused by multiple mechanisms, including altered neuronal morphology and neuronal loss. For example, postmortem OB tissue of PD patients reveals substantial loss of ventral glomerular areas in the OB, correlated with phosphorylated *α*-synuclein load (Zapiec et al., 2017). This α-synuclein accumulation specifically induces apoptosis of DA neurons (Xu et al., 2002), likely contributing to the reduced size or number of glomeruli in the OB. In AD, the accumulation of P-tau and Aβ drives neuronal atrophy throughout the brain, including M/TCs in the OB (Struble and Clark, 1992; Yao et al., 2017; Li et al., 2019b). This aligns with studies showing that the MC layer is predominantly affected by tau pathology in an AD mouse model (Yang et al., 2016). While reduced OB volume is also common in SCZ, direct evidence for altered neuronal morphology in OB is limited for neurodevelopmental disorders. However, it was recently shown that a mouse model of OE inflammation which closely mimics inflammatory processes in the OE of first-episode psychosis patients shows reduced glomerular size and OB volume, alongside decreased numbers of OSNs and M/TCs (Yang et al., 2024). Further, animal models of SCZ, such as immune-challenged DISC1 knockdown mice and 22q11-deletion mice, show reduced soma size and dendritic arborization of pyramidal neurons in brain regions such as LEC, HP, and PFC (Chini et al., 2020; Kringel et al., 2023; Stark et al., 2008; Fénelon et al., 2013). However, so far it is unknown whether the same holds for M/TCs, which strongly express DISC (Schurov et al., 2004). Impaired neurogenesis might also contribute to OB volume reduction in both neurodegenerative and neurodevelopmental disorders. Neuroblasts generated in the subventricular zone (SVZ) continuously migrate to the OB, where they differentiate into GCs and PGCs (Belluzzi et al., 2003; Livneh et al., 2014). Impairments of adult SVZ neurogenesis are evident early in animal models of AD and PD (Winner et al., 2008, 2011; Rodríguez et al., 2009; Scopa et al., 2020; Esteve et al., 2022; Martin-Lopez et al., 2023). Similarly, disruptions in SVZ neurogenesis are seen in neurodevelopmental disorders. DISC1 knockdown leads to reduced progenitor cell proliferation in the SVZ during embryonic stages (Mao et al., 2009). Additionally, MIA leads to altered proliferation in the SVZ of neonatal mice and further contributes to reduced adult neurogenesis in the OB (Liu et al., 2013b; Loayza et al., 2023).

Aside from structural changes, alterations in the neuronal activity within olfactory circuits are common in these disorders. OSNs, the first neurons to receive odor information, are reduced in numbers and show smaller odor-evoked responses in AD, resulting in diminished excitatory input to the OB (Chen et al., 2021b). This, combined with reduced dendritic spine density in GCs and impaired dendro-dendritic inhibition onto MCs, leads to increased gamma-band OB network activity (Wesson et al., 2011; Chen et al., 2021b; Li et al., 2019a, 2019b). Similarly, overexpression of α-synuclein in the OB, one of the key features of PD, leads to reduced GC activity and impaired dendro-dendritic inhibition onto MCs, along with elevated odor-evoked gamma oscillations (Chen et al., 2021a). In neuropsychiatric disorders, a broadband reduction of oscillatory power in the OBs of a SCZ mouse model was accompanied by reduced firing of M/TCs (Parbst et al., 2024). Furthermore, compromised functional connectivity between brain regions accounting for olfactory and cognitive processing is evident in both neurodegenerative and neurodevelopmental disorders. For instance, patients with SCZ show reduced functional connectivity between PIR, PFC, and nucleus accumbens (Kiparizoska and Ikuta, 2017) as well as between the HP and PFC (Adams et al., 2020). In AD, disruption of functional connectivity between olfactory networks (including PIR and OFC) and HP is linked to cognitive decline (Lu et al., 2019a, 2019b). Already during neonatal development, desynchronization between LEC, HP, and PFC manifests in an animal model of neuropsychiatric disorders such as SCZ (Hartung et al., 2016; Xu et al., 2021). A recent study showed that functional connectivity between OB and HP, as well as, OB and PFC was significantly reduced in the same animal model (Parbst et al., 2024). Similarly, in animal models of ASD, reduced OB activity and altered connectivity between HP and PFC have been reported (Cheaha et al., 2015; Richter et al., 2019). During early development, olfactory inputs are critical in synchronizing brain regions involved in olfactory and cognitive processing (Gretenkord et al., 2019; Kostka and Hanganu-Opatz, 2023). Notably, silencing M/TC activity in the OB during early development impairs the maturation of olfactory-hippocampal networks and cognitive abilities later in life (Chen et al., 2023). These findings suggest that altered olfactory activity can disrupt the development of functional coupling within neuronal networks, potentially contributing to cognitive impairments, seen in many neurodegenerative and neurodevelopmental disorders (Bennetto et al., 1996; Jahn, 2013; Davis and Racette, 2016; Guo et al., 2019).

Alterations in neurotransmitter systems, particularly the DA system, also significantly contribute to olfactory deficits in neurodegenerative and neurodevelopmental disorders. DA neurons in the OB inhibit olfactory transmission in the olfactory glomeruli (Wilson and Sullivan, 1995; Hsia et al., 1999) and are important for the encoding of innate odor values (Kato et al., 2023). In PD and AD, loss of DA neurons in the substantia nigra and ventral tegmental area leads to impaired DA outflow to several brain areas, including the OB (German et al., 1989; Nobili et al., 2017). Interestingly, increased numbers of DA neurons have been observed in the OBs of PD and AD patients (Huisman et al., 2004; Mundiñano et al., 2011). Neurodevelopmental disorders, such as ASD, also exhibit altered DA signaling and DA receptor abnormalities (Pavăl, 2017; Kosillo and Bateup, 2021; Pavăl and Micluția, 2021). Beyond DA, cholinergic transmission plays an important role in olfactory processing and is frequently altered in these disorders (Doty, 2017). For example, acetylcholine dysfunction exacerbates Aβ pathology in AD and cholinergic receptor abnormalities are present in ASD (Gil-Bea et al., 2012; Ovsepian et al., 2019; Vallés and Barrantes, 2021).

Overall, several intertwined mechanisms, such as neuronal loss, reduced neurogenesis, impaired synaptic transmission, and altered neurotransmitter signaling can lead to structural and functional alterations in olfactory circuits, contributing to the olfactory deficits characteristic of both neurodevelopmental and neurodegenerative diseases.

## Conclusion

Olfactory deficits emerge early, often preceding the clinical diagnosis of neurodegenerative and neurodevelopmental disorders. Whether this relationship is causal or merely an epiphenomenon remains an open question. However, the presence of olfactory dysfunction and alterations in olfactory-related brain areas well before cognitive and motor symptoms suggest a potential causal relationship. Notably, individuals at high risk for psychiatric disorders, such as relatives of SCZ patients, often exhibit olfactory impairments, indicating that these deficits are unlikely due to secondary effects of treatment (Turetsky et al., 2018).

The OE as well as primary olfactory areas such as OB and AON often show pathological changes in prodromal disease stages before the involvement of other brain areas. In line with the *α*-synuclein transmission hypothesis (McCann et al., 2016) injection of human α-synuclein fibrils into the OBs of young mice leads to a spread of α-synuclein aggregates across several brain regions, correlating with increasing olfactory deficits (Rey et al., 2016). Similarly, the injection of soluble Aβ in an AD mouse model shows similar spreading patterns (He et al., 2018). This suggests that in neurodegenerative diseases, pathological aggregation of proteins originates in olfactory areas and spreads in a prion-like manner to higher-order cortical regions, contributing to disease progression. Moreover, in both neurodegenerative and neurodevelopmental disorders, olfactory dysfunction is linked to reduced functional connectivity with downstream brain regions, potentially accelerating cognitive decline. For example, layer 2 neurons in LEC, which receive direct OB input and project to HP, are especially vulnerable, showing functional and morphological alterations in AD (Stranahan and Mattson, 2010). Thus disrupted inputs from the OB may, cause structural and functional changes along olfactory pathways. Supporting this, studies have shown that recently acquired sensory loss can alter both morphology and functional connectivity between the PIR and higher-order cortical brain regions (Bitter et al., 2010; Iravani et al., 2021). Thus, olfactory circuits may play a dual role: they could serve as a route for the spread of pathogenic proteins to downstream brain areas, and disruptions in olfactory processing in the OE and OB could have lasting consequences on higher-order cortical regions, potentially contributing to cognitive deficits.

On the other hand, olfactory impairments might coincide with disease progression or result from secondary effects. For example, disruptions in forebrain development and altered neurotransmitter signaling can lead to olfactory dysfunctions (Doty, 2017). Furthermore, the propagation of tau, from the temporal lobe to olfactory circuits was shown to drive the degradation of odor perception as individuals get older (Diez et al., 2024). Moreover, while many patients with neurodevelopmental and neurodegenerative disorders experience impaired olfaction, this is not universal, suggesting that olfactory system involvement is not a necessary feature of disease progression in all cases.

Regardless of whether olfactory dysfunction is a cause or consequence of these diseases, it consistently occurs early, often before a clinical diagnosis is made. Testing olfactory abilities for example with simple Sniffn’ Sticks tests or the University of Pennsylvania Smell Identification Test is an effective and inexpensive way to identify individuals with olfactory deficits. Since reliable biomarkers for early diagnostics are lacking, monitoring olfactory deficits in individuals at risk or incorporating olfactory testing into routine health checks has great potential (Dan et al., 2021). In addition, olfactory testing may serve as a tool for monitoring disease progression or evaluating therapeutic effects (Berendse et al., 2011). Further research is necessary to understand the mechanisms underlying olfactory dysfunctions, as this could offer valuable insights into the etiology and progression of these diseases.
